# Relationship between *Helicobacter pylori* and Rosacea: review and discussion

**DOI:** 10.1186/s12879-018-3232-4

**Published:** 2018-07-11

**Authors:** Xingzhe Yang

**Affiliations:** Beijing University of Chinese Medicine, 11 Bei San Huan Dong Lu, Chaoyang District, Beijing, 100029 China

**Keywords:** Rosacea, *Helicobacter pylori*, Related, Correlation, Epidemiological investigation, Experiment, Anti-*H. pylori* therapy, Mechanism

## Abstract

**Background:**

Rosacea is an inflammatory disease affecting the central part of face characterized by persistent or recurrent episodes of erythema, papules, pustules and telangiectasias of unknown etiology. *Helicobacter pylori (H. pylori)* is a gram-negative bacillus, which is one of the main causes of chronic gastritis, gastric cancer and gastrointestinal ulcers. Recent evidences have suggested that *H. pylori* infection is closely related to the occurrence of diseases*.* In recent years, studies have found that *Helicobacter pylori* infection is associated with the occurrence of acne rosacea. So the treatment of *Helicobacter pylori* infection may be a therapeutic method of acne rosacea. But it continues to be controversial. In other studies, the treatment of *Helicobacter pylori* did not significantly reduce the severity of acne rosacea. To further explore the association between acne rosacea and *Helicobacter pylori* infection, a summarize method was used to study the relationship between acne rosacea and *Helicobacter pylori,* providing reference for clinical acne rosacea therapy.

**Methods:**

Systematic searches were conducted on Wanfang Data, CQVIP, Springer, Public Health Management Corporation (PHMC), CNKI, and Pubmed, from January 1,2008 to Mar. 1, 2018, using *Helicobacter pylori* and rosacea to retrieve the literature. Depending on the inclusion and exclusion criteria, 27 articles considered or confirmed the correlation between *H. pylori* and rosacea.

**Results:**

Epidemiological investigations and experiments have confirmed that *H. pylori* infection is associated with the development of rosacea. The effect of anti-*H. pylori* therapy is better than the routine therapy for rosacea. *H. pylori* can stimulate the immune system to produce a large number of inflammatory mediators, leading to the occurrence and aggravation of rosacea inflammation.

**Conclusions:**

It is confirmed that *H. pylori* infection is involved in the development of rosacea. It is suggested that rosacea patients should be tested for *H. pylori* infection, the *H. pylori*-positive rosacea patients should be treated with eradication of *H. pylori*, so as to enhance the therapeutic effect of rosacea*.* This study adds that *H. pylori* infection is involved in the development of rosacea. Epidemiological investigations and experiments have confirmed the rationality. The effect of anti-*H. pylori* therapy is better than the routine therapy for rosacea. *H. pylori-*positive rosacea patients should be treated with the therapeutic method of eradication of *H. pylori.*

## Background

Rosacea is an inflammatory disease of unknown etiology, the role of *H. pylori* infection factor in the pathogenesis of rosacea has been paying close attention to epidemiological, experimental and clinical aspects of *H. pylori,* which has been confirmed that *H. pylori* infection is associated with the development of rosacea. But the data are limited, and further clinical and laboratory researches are required to assess the actual existence and relevance of many purported associations. That’s already known about this topic which rosacea is an inflammatory disease affecting the central part of face of unknown etiology, affecting 12.3% Russians and 5.0% Germans and 2.0% ~ 2.3% Americans. As a gram-negative bacillus, *H. pylori* infection is closely related to the occurrence of diseases. This study adds that *H. pylori* infection is involved in the development of rosacea. Epidemiological investigations and experiments have confirmed the rationality. The effect of anti-*H. pylori* therapy is better than the routine therapy for rosacea. *H. pylori*-positive rosacea patients should be interpreted with eradication of *H. pylori.* Relationship between acne rosacea and *Helicobacter pylori* was studied*,* in order *to* provide reference for clinical acne rosacea therapy.

## Methods

Systematic searches were conducted on Wanfang Data, CQVIP, Springer, Public Health Management Corporation (PHMC), CNKI, and Pubmed, from January 1st 2008 up to now, using *Helicobacter pylori* and rosacea to retrieve the literature. The total number is 247:19 in Wanfang, 4 in CQVIP, 0 in Springer, 121 in PHMC, 2 in CNKI and 101 in Pubmed. Depending on the inclusion and exclusion criteria, 27 articles considered or confirmed the correlation between *H. pylori* and rosacea.

## Results

**Fig. 1:**
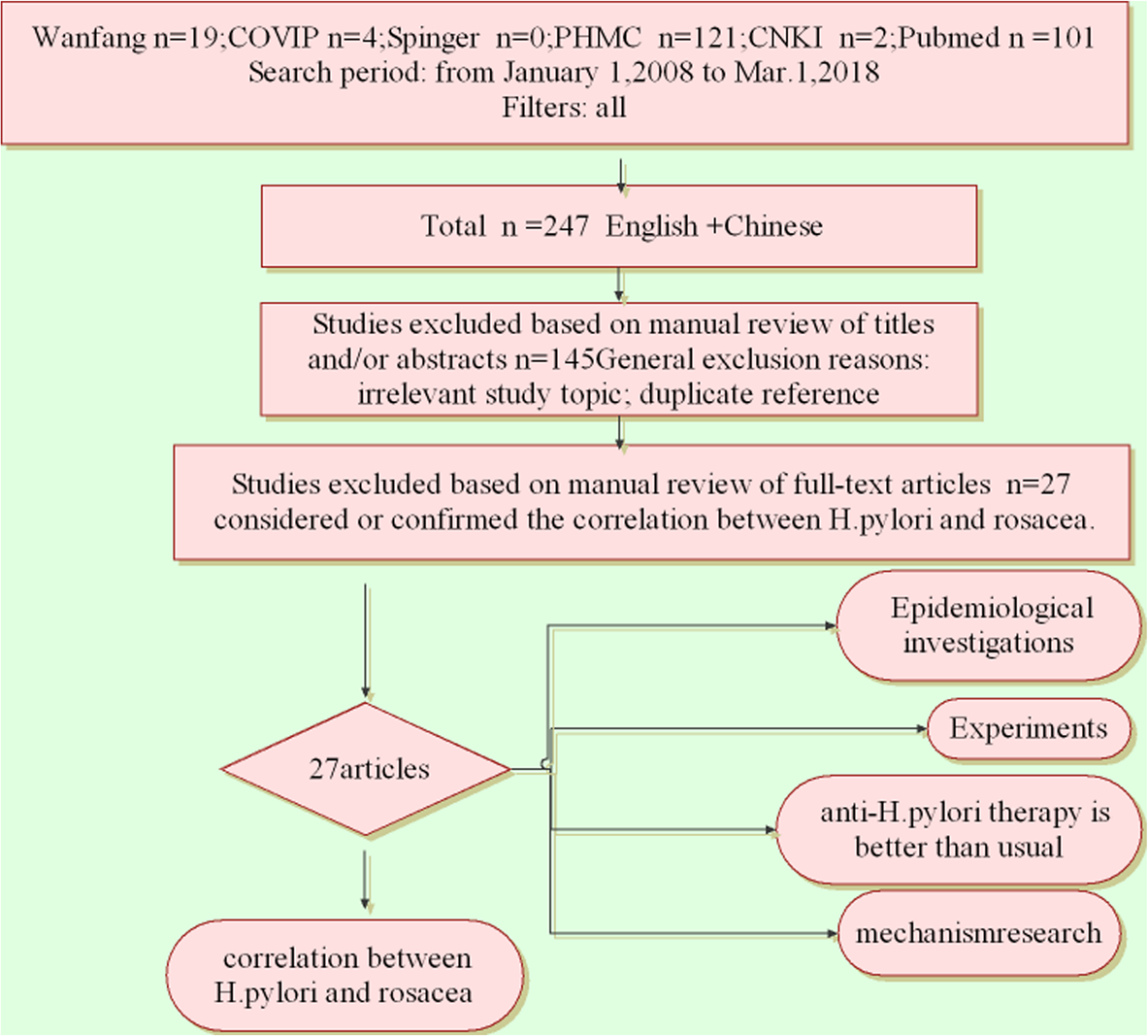
Preferred reporting items for *H. pylori* and rosacea. Systematic searches were conducted in Wanfang Data, CQVIP, Springer, Public Health Management Corporation (PHMC), CNKI and Pubmed, from January 1st 2008 to Mar. 1, 2018, using Helicobacter pylori and rosacea to retrieve the literature. The total is 247:19 in Wanfang, 4 in CQVIP, 0 in Springer, 121 in PHMC, 2 in CNKI and 101 in Pubmed. According to the inclusion and exclusion criteria, 27 articles considered or confirmed the correlation between *H. pylori* and rosacea.

More studies have shown that *H. pylori* is involved in the occurrence and development of Rosacea In recent years [1, 2]. Systematic researches were conducted on Wanfang Data, CQVIP, Springer, Public Health Management Corporation (PHMC), CNKI, and Pubmed, from January 1st 2008 up to now, using *Helicobacter pylori* and rosacea to retrieve the literature. The total number is 247:19 in Wanfang, 4 in CQVIP, 0 in Springer, 121 in PHMC, 2 in CNKI and 101 in Pubmed. Depending on the inclusion and exclusion criteria, 27 articles considered or confirmed the correlation between *H. pylori* and rosacea (Fig. 1)*.*

## Discussion

Rosacea is an inflammatory disease affecting the central part of face characterized by persistent or recurrent episodes of erythema, papules, papulo pustules and telangiectasias of unknown etiology [3]. It is divided into erythematotelangiectatic rosacea (ETR), papulopustular rosacea (PPR), phymatous rosacea (PHR) and ocular rosacea (OR) by American National Rosacea Society Expert Committee (NRSEC) [4] and Chinese Consensus on Diagnosis and Treatment of Rosacea 2016 [5]. Incidences of rosacea in Russia and Germany were 12.3 and 5.0% [6], and 2.0% ~ 2.3% [7] in the United States. The pathogenesis of rosacea is unclear and it may be caused by multifactorial chronic inflammation, which is dominated by innate immunity and abnormal vasomotor function [5].

*Helicobacter pylori *(*H. pylori)* is a gram-negative bacillus, which is one of the main causes of chronic gastritis, gastric cancer and gastrointestinal ulcers. Recent evidences have suggested that *H. pylori* infection played a role in the pathogenesis of a variety of skin diseases [8]. Egeberg A [9] performed a nationwide cohort study. A total of 49,475 rosacea patients and 4,312,213 general population controls were identified using nationwide administrative registers*.* Rosacea is associated with certain gastrointestinal diseases, but the possible pathogenic relevance is unknown.

### Epidemiological investigations have confirmed that *H. pylori* infection is associated with the development of rosacea

Liu YF studied 50 rosacea patients [10] whose positive rate of *H. pylori* was significantly higher than that of healthy people. The efficacy of anti *H. pylori* treatment is much better than conventional treatment*.* It indicated that there is a certain relationship between Mongolian *H. pylori* and rosacea in the Inner Mongolia area.

Jørgensen AR found weak associations between rosacea and *Helicobacter pylori* infection as well as an effect of *Helicobacter pylori* therapy for rosacea symptoms, albeit that did not reach statistical significance. But analysis restricted to C-urea-breath test showed a significant association (OR 3.12, 95% CI 1.92–5.0, *p* < 0.0001) [11]. Talebi Bezmin Abadi A assured the success of standard triple therapy to eradicate the bacterium because of a high rate of antibiotic resistance, and a better antibiotic therapy in the battle against *H. pylori* is needed. It needs further analysis before being able to change the current universal or even national guidelines in the treatment of any *H. pylori*-infected patients [12]. Yu JW studied the infestation rate of *Helicobacter pylori* and factors which had affected the infestation in the Inner Mongolian border recruits [13]. According to the consensus opinion of Maastricht-IV [14], Nine hundred Recruits aged 16 to 24 were enrolled in this cross-sectional study*. H. pylori* infection was detected by 13C-urea breath test, and the related risk factors were surveyed by using questionnaires*.* Logistic regression analysis revealed that halitosis with acne were positively correlated with *H. pylori.* The *H. pylori* infection rate of the recruits with acne was 47*.*2%, which was higher than that of the recruits without acne whose rate was 46.8%. But the difference was not statistically significant (x^2^ = 0*.*77, *P* = 0*.*381). The *H. pylori* infection rate in recruits with oral odor and acne was 52*.*7%, significantly higher than that in recruits without oral odor and acne whose rate was 39.7% (x^2^ = 18.96, *P* = 0.008).

Wang AS [15] studied relationships between Helicobacter Pylori (*Helicobacter Pylori*)Infection and Acne Vulgaris in 980 cases of College Students, *H. pylori* was detected in 980 college students with acne and 260 college students without acne. The results showed that the H. pylori (*H. pylori*) positive rate of the acne group was 72.45%. The positive rate of H. pylori(*H. pylori*) was 39.23% in non-acne group. The difference was statistically significant (x^2^ = 100.3, *P* < 0.0001).

### Experiments show correlations between *H. pylori* infection and rosacea

Yuan XR studied 42 rosacea patients and 33 acne vulgaris patients [16]. The level of serum specific IgG antibodies against *H. pylori* was determined, and the gastrointestinal symptoms of the patients were studied*.* 20 rosacea patients received the standard anti-H. pylori triple chemotherapy. The results showed that the serologic anti-*H. pylori* IgG level of the rosacea patients was higher than those of the controls, and gastrointestinal dysfunction was more frequent than those in the controls*.* The efficacy of anti-*H. pylori* therapy was also significantly better than usual. It indicates that *H. pylori* infection may be important in rosacea*.*

Szlachcic A [17] studied the relevance between *H. pylori* infection and rosacea. It concluded that *H. pylori* is closely connected with some digestive tract diseases and also the occurrence of some extra-gastrointestinal diseases. Studies confirmed the link between *H. pylori* infection and rosacea. The reason is perhaps that the toxic factor of *H. pylori* may cause delayed skin changes. It may be also possible that *H. pylori* directly induces complement activation and results in skin changes [18]. *H. pylori* can induce skin inflammation through two mechanisms. Firstly, *H. pylori* can affect skin physiological activities by increasing the concentration of nitrous oxide (N_2_O), Such as vasodilation, inflammation mediated and immune stimulation. Furthermore, *H. pylori* infection can also induce a specific cytotoxic reaction, through which can express cytotoxic genes A (cytotoxin-associated gene A, cagA), TNF-α and IL-8, and then induce a series of inflammatory reactions. *H. pylori* eradication can significantly alleviate the symptoms of rosacea, especially the subtype of pimples of papules. It wishes to point out a new direction for the treatment of rosacea patients. Bhattarai S’s study of the prevalence of *H. pylori* in 26 rosacea patients indicates that *H. pylori* infection is positively correlated with rosacea [19].

Hong J observed detection rate of *H. pylori* Urase-IgG and *H. pylori* CagA-IgG in peripheral blood of 39 rosacea patients [20], the results showed that the total incidences of *H. pylori* Urase-IgG and *H. pylori* CagA-IgG in all 39 rosacea patients were not high, but the incidence rates in rosacea patients with digestive tract symptoms were 86*.*7% in *H. pylori* Urase-IgG and 80% in *H. pylori* CagA-IgG, which were significantly higher than that in rosacea patients without alimentary tract symptoms whose rates were 16*.*7 and 0%. The results suggested that the virulent strain of *H. pylori*, especially the strain of *H. pylori*, may be involved in the occurrence and development of digestive tract symptoms and skin papules injury in rosacea*.* The results of El-khalawany M [1] showed that the positive rate of *H. pylori* in rosacea patients was significantly higher than that in normal controls, and the *H. pylori* infection rate in rosacea patients with dyspepsia was higher than that in patients with papular pustules,and that in patients with papular pustules was higher than that in patients with anectasis*.*

### The effect of anti-*H. pylori* therapy is better than the routine therapy for rosacea

In patients with peripheral the lesions showed erythema, papules, pustule and possibly accompanied by gastrointestinal discomfort*.* The 13C-urea breath test for *Helicobacter pylori* screening was a good thing*.* After systemic anti**-***H. pylori* (including PPI + 2 antibiotics Bismuth) topical drug therapy and medical skin care products to repair the skin barrier,the results showed that perioral rosacea was associated with H. pylori infection in the digestive tract. *H. pylori* was discovered in 84.1, and 61.4% of patients who presented with digestive symptoms, which denote the relation between rosacea and digestive problems which confirm the study results of Sharma et al. [21] Rosacea is a skin disease with an obscure and complicated pathogenesis. Numerous mechanisms have been described, but its etiology remains an enigma. There is inadequate evidence regarding how determinant the role of *H. pylori* is. Built on the fact that the studies were not extensive, controlled studies are required [22]. The cure rates of *H. pylori* in rosacea patients and controls were 80% (16/20) and 85% (17/20), respectively [23].

Zhang HY [24] observed the therapeutic effect and mechanism of sequential therapy with the combination between Chinese and western medicine of *H. pylori-*positive acne rosacea (AR) with spleen-stomach dampness heat*.* Sixty qualified patients with spleen-stomach dampness, heat was equally randomized into a treatment group and control group*.* The clinical effective rate in the treatment group was significantly better than that in the control group (*P* < 0*.*05). The *H. pylori*-positive rate, concentrations of IL-8 and TNF-α in serum was lower in the treatment group than in the previous treatment group, and the decrease was superior to control group (*P* < 0*.*01)*.* AR has a certain relationship with *H. pylori*-positive rate*.* The sequential therapy with the combination between Chinese and western medicine could effectively relieve the clinical symptoms of *H. pylori*-positive AR patients with spleen-stomach dampness-heat, and the mechanism of which is probably related to the decrease of *H. pylori-*positive rate and serum levels of IL-8 and TNF-α.

### Discussion

Since 1999, when Szlachcics A [25] first proposed that rosacea infection was related to *Helicobacter pylori* infection, the role of *H. pylori* infection factors in the pathogenesis of rosacea has been paying close attention to the epidemiological, experimental and clinical aspects of *Helicobacter pylori*, which have been suggested or confirmed that *Helicobacter pylori* infection is associated with the development of rosacea*.* The following studies illustrate the mechanism of *Helicobacter pylori* infection in relation to rosacea from the perspective of inflammation and genes.

It has been found that *H. pylori* infection is closely linked to the occurrence of diseases. *H. pylori* can stimulate the immune system to produce a large number of inflammatory mediators, leading to the occurrence and aggravation of rosacea inflammation [26]. As one of the infectious factors of rosacea [27], Extradermal bacteria, such as small intestinal bacteria, *H. pylori* can cause or exacerbate rosacea by producing large amounts of cytokines, especially papular pustules (PPR) [28].

At present, there are two mechanisms of skin inflammations induced by *H. pylori.* Firstly, *H. pylori* can affect skin physiological activities by increasing the concentration of Nitrous Oxide in vivo, such as vasodilation, inflammation and immune stimulation*.* Furthermore, *H. pylori* infection can also induce a specific cytotoxic reaction, which can express cytotoxic related genes A (cytotoxin-associated gene A, CagA), TNF-α and IL-8, and cause a series of inflammatory reactions [19].

From the perspective of gene research, Wang WW [29] studied the relationship between gene polymorphism and susceptibility to *H. pylori.* It concluded that -251A/T polymorphism of IL-8 gene is closely related to the susceptibility of T Alleles which may be a risk factor for *H. pylori* infection.

Zhang Y [30] did the research on *H. pylori* gene in gastric mucosa of patients with rosacea. The expression of *H. pylori* gene in gastric mucosa of patients with different pathological types of rosacea is different, while rosacea is probably one of the symptoms caused by inflammatory mediators of IL-8 and IL-1 which induced by *H. pylori* virulence gene. But further clinical and laboratory researches are required to assess the actual existence and relevance of various purported associations.

**Fig. 2:**
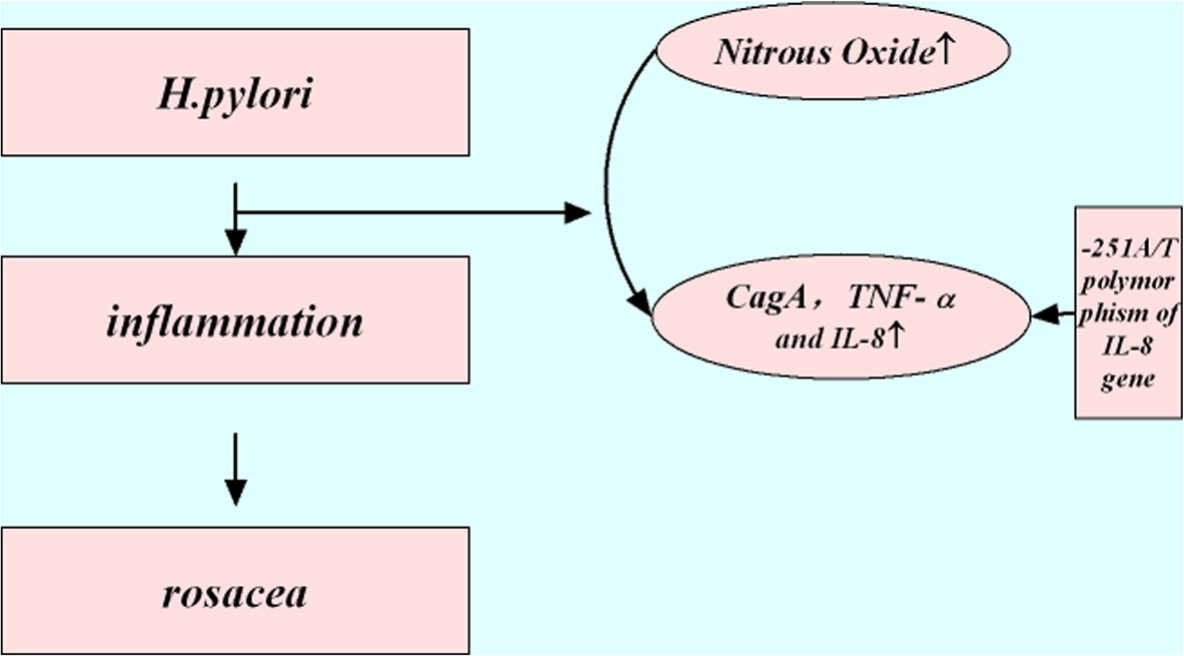
Mechanism of *H. pylori* infection in relation to rosacea. The sequential therapy with the combination between Chinese and western medicine could effectively relieve the clinical symptoms of *H. pylori-*positive AR patients with spleen-stomach dampness-heat, and the mechanism of which is probably related to the decrease of *H. pylori* -positive rate and serum levels of IL- 8 and TNF- α

There is a definite relationship between *H. pylori* and the occurrence and development of rosacea*.* It is speculated that *H. pylori* infection may play a role in the development of rosacea in diverse ethnic groups and may be an etiology of rosacea*.* Of course, as a multi-stage disease, the occurrence and development of the resource may not be determined only by one factor which may promote or play a decisive role in the development of the disease*.* Therefore, it is suggested that rosacea patients should be tested for *H. pylori* infection, the *H. pylori*-positive rosacea patients should be treated with eradication of *H. pylori*, so as to enhance the therapeutic effect of rosacea (Fig. 2)*.*

## Conclusions

*H. pylori* infection is associated with the development of rosacea. The effect of anti-*H. pylori* therapy is better than the routine therapy for rosacea. *H. pylori*-positive rosacea patients should be treated with eradication of *H. pylori*.

## Abbreviations

Anti-*H. pylori*: Anti *Helicobacter pylori*
H. pylori.*(H. pylori)*: Helicobacter pylori(*Helicobacter pylor*i)
*H. pylori*-positive: *Helicobacter pylori* positive
